# Retroperitoneal Ectopic Location of an Intrauterine Device Revealed by Renal Colic: An Exceptional Case

**DOI:** 10.1155/2020/8893750

**Published:** 2020-08-14

**Authors:** Idriss Ziani, Imad Boualaoui, Ahmed Ibrahimi, Hachem El Sayegh, Lounis Benslimane, Yassine Nouini

**Affiliations:** ^1^Department of Urological Surgery A, University Hospital of Rabat, Rabat, Morocco; ^2^Faculty of Medicine-Mohammed V University RABAT, Morocco

## Abstract

The intrauterine device (IUD) is one of the most effective contraceptive methods. Its Pearl Index is less than 1 per 100 women. It is the most used method around the world: about 100 million users. However, its insertion can cause certain complications, such as infection, expulsion, or perforation essentially when the rules of use are poorly applied. Perforation remains exceptional but one of the most serious complications. Indeed, after a perforation, the IUD could be located in different neighboring organs. We report a new case of IUD ectopic location in the peritoneal cavity, which was diagnosed 7 years after the insertion and as part of the renal colic assessment. The surgery was performed to remove the IUD which was embedded in the peritoneum and compresses the ureter and causes dilation upstream. To our knowledge, this is the first case reported in the literature of an ectopic location in the retroperitoneal space of an intrauterine device.

## 1. Introduction

The intrauterine device (IUD) is one of the most effective methods of contraception. Its Pearl Index is less than 1 per 100 women [1]. It is the most widely used method around the world: about 100 million users. However, its insertion can cause some complications, such as infection, expulsion, or perforation essentially when the rules of use are poorly applied. The perforation remains exceptional but one of the most serious complications. Indeed, after a perforation, the IUD may be located in different neighboring organs. Ectopic locations at the Douglas pouch, omentum, mesentery, colon, and bladder have been described [2], often with lithiasic complications in the bladder [3].

We report a new case of IUD extralocation in the retroperitoneal cavity, which was diagnosed 7 years after the insertion and as part of the renal colic assessment. The surgery was performed to remove the IUD which was embedded in the peritoneum compressing the ureter and causing dilation upstream.

## 2. Patient and Observation

Mrs. A.k., 29 years old, is a primigravida by caesarean delivery with no significant pathological history and known carrier of a copper T-uterine device inserted 7 years earlier. The first control one month after insertion revealed no abnormalities. A second check 6 months after the observation by the patient of the disappearance of nylon thread and clinical examination shows an empty uterine cavity. No radiological investigation was considered at the time.

**Figure 1:**
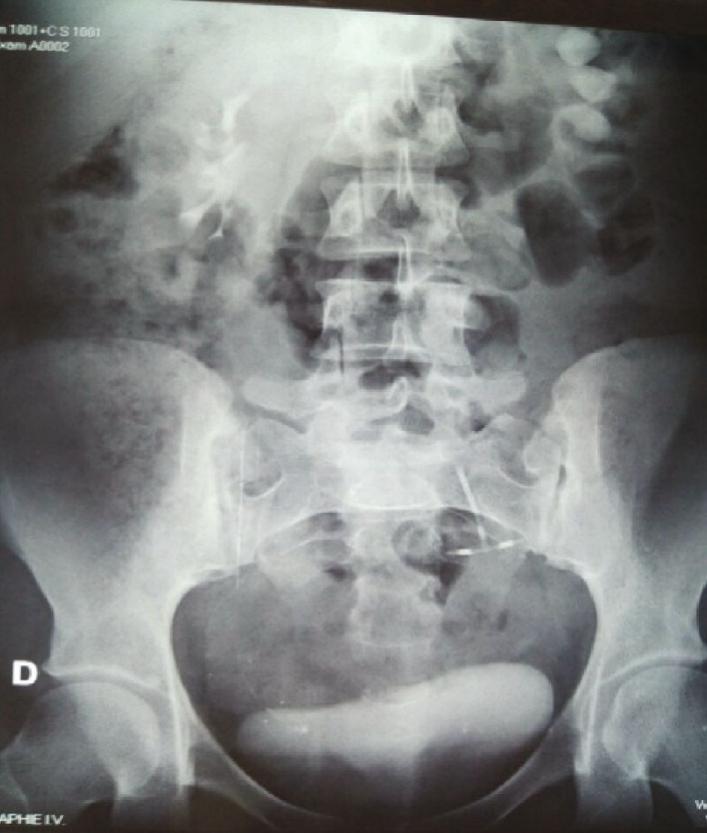
ASP while standing with an IUD at the pelvic area of the uterine.

**Figure 2:**
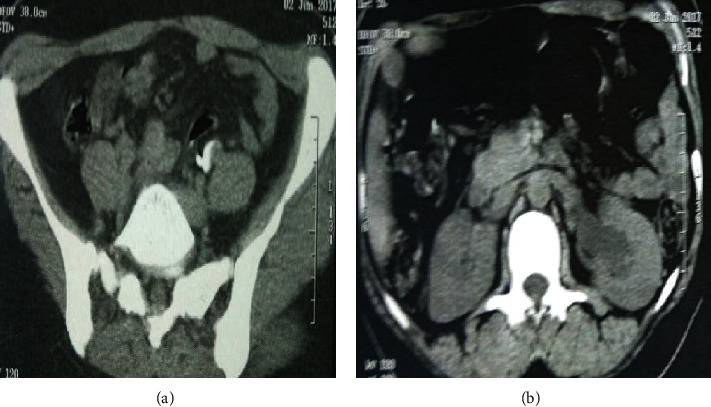
CT image (cross section). (a) CT image showing the retroperitoneal location with left ureteral compression. (b) CT image showing left ureterohydronephrosis secondary to ureteral compression by the IUD.

**Figure 3:**
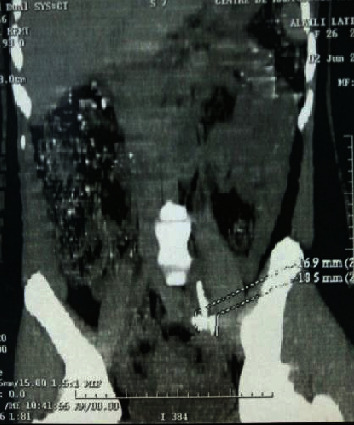
CT scan sagittal objective retroperitoneal localization of the IUD.

**Figure 4:**
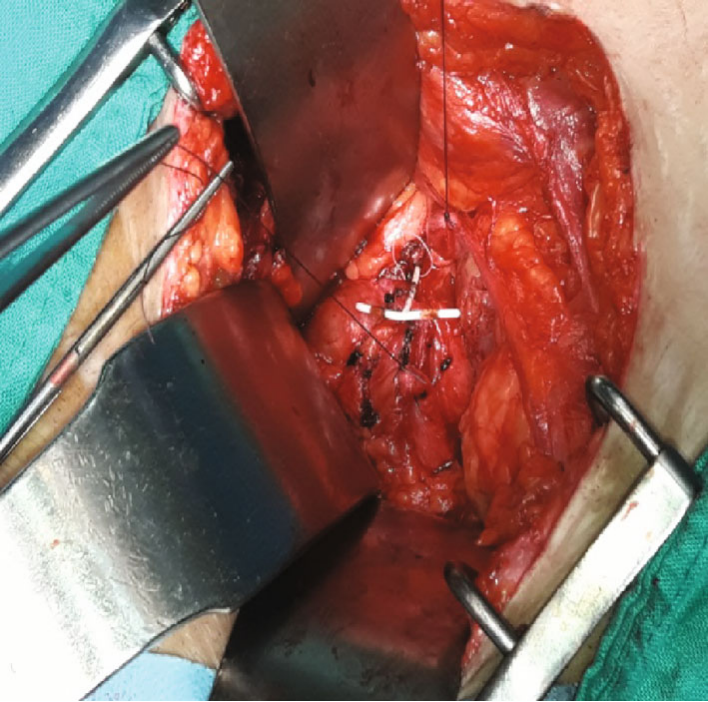
The IUD which vertical limb was embedded in the left ureter (intraoperative image).

**Figure 5:**
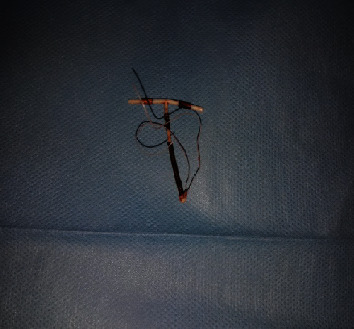
The IUD after surgical extraction.

She consults for renal colic evolving since one year treated symptomatically by analgesic treatment; before the recurrence and the persistence of the symptomatology the patient consulted in our formation. The clinical examination found the patient to be in good general condition; the abdomen is supple with a slight sensitivity to palpation of the left lumbar fossa with no observation of IUD strings in the speculum examination. The vaginal examination shows a normal-sized uterus. An initial radiological assessment included an abdominal X-ray which reveals an extralocation of IUD in the left ureter path (Figure 1) and ultrasound that shows a left ureterohydronephrosis (renal pelvis at 25 mm). The abdominal CT confirms the retroperitoneal location and the left ureteral compression by the IUD (Figures 2 and 3). The diagnosis of a migratory IUD is thus retained. Given her surgical history, the patient had laparotomy for IUD removal. Surgical exploration revealed parietal adhesions that were gently released, allowing the observation of the extralocated IUD, whose vertical limb was embedded in the left ureter. The IUD was removed without difficulty after resection anastomosis of the ureter made with placement of a double J probe. No complication was noted (Figures 4 and 5).

## 3. Discussion

The IUD is one of the most used means for nondefinitive contraception in the world [1, 4, 5]. Like any foreign body, the IUD can have risks and complications, such as migration after uterine perforation that remains rare and infection [6]. This migration can take several directions; it is usually in the peritoneal cavity and rarely in the neighboring pelvic organs, mainly the bladder and the rectosigmoid [1, 5, 7]. Pelvic locations out of the bladder are exceptional [1, 4]. Ibghi et al. reported stenosis of the iliac vein secondary to the migration of an IUD [8]. According to our knowledge, this observation is the first case of retroperitoneal migration with ureter insertion reported in the literature.

The copper in some IUDs can trigger an inflammatory reaction leading to a contraceptive effect, but it can also be involved in the process of long-term perforation and uterine transmigration [9]. Intramyometrial migration begins with the incarceration of an IUD branch in the myometrium, and inflammatory phenomena as well as uterine contractions will allow the IUD to continue its migration [1, 6, 7]. Another study explains that the IUD is driven to abnormal sites by uterine forces. In fact, Goldstuck and Wildemeersch showed that the nature of perforation may be primary, secondary, or both. They also suggested that the forces of myometrial contractions are leading factors in IUD moving out of the uterine cavity. Moreover, they bring to light the crucial effect of force vector direction and that the summated multivectorial force determines the IUD direction [10]. Factors predisposed to this extralocation are especially a weakening of the myometrium by multiple pregnancies and caesareans, ante- or retroverted and hypoplastic uterus, and the laying of IUD too early in the postpartum period [1, 4]. The delay of this complication, which would correspond to the time between the insertion of the IUD and the appearance of the first clinical signs, can be several years (up to 36 years) [1].

Clinically, the symptomatology is variable according to the site of ectopic location and the type of IUD [10, 11]; in our case, the main symptom was renal colic because of the ureteral compression and the insertion of a branch of IUD in the ureter as well as periureteral fibrosis which was responsible for ureteral stenosis.

Thus, uterine perforation by IUD is usually asymptomatic, except when it occurs primarily at the time of the pose, resulting in violent pain, which must attract the attention of the doctor. On examination, the perforation is suspected before the disappearance of the reference thread, after ensuring that this landmark is not raised in the endocervix, and this is the case of our patient whose clinical examination shows the lack of thread in the vulva and endocervix [10–12].

The means to locate an extrauterine IUD will be ultrasound and, if unsuccessful, abdominal X-ray to look for it in the abdomen before concluding to an unknown expulsion. Computed tomography (CT) or magnetic resonance imaging will locate it precisely. When migrating, the IUD can be localized in the Douglas pouch, the broad ligament, and the omentum (45%) [13, 12]. Digestive localizations (such as the mesentery and the colon) and the bladder are less frequent [2]; no case of retroperitoneal migration has been diagnosed in the literature.

In our observation, the diagnosis was confirmed by the spiral CT with reconstruction which allows a better study of the repercussion on the upper urinary tract and specifies the exact location of the ectopic IUD.

The majority of authors believe that the removal of the device is essential given the risk of digestive and especially urological complications like the case we discussed. The removal of the migratory IUD is most often done by laparoscopy. In the literature, its success rate varies between 44 and 100% [13, 14], depending on the number of cases treated, the location of the IUD, and the experience of the operator. In our observation, we recommended a laparotomy upon seeing the patient's surgical history.

## 4. Conclusion

The IUD is an effective contraceptive method; its insertion is a simple medical procedure that requires minimum knowledge and experience. Perforation associated with retroperitoneal ectopic location is one of the rarest and most dangerous complications. Laparoscopic or even laparotomic removal of the IUD is essential to avoid ureteral perforation and the repercussions on the upper urinary tract.

## Abbreviations

IUD: Intrauterine device
CT: Computed tomography.
